# Supplementary material for: Prevalence and predictors of antibiotic prescription among patients hospitalized with viral lower respiratory tract infections in Southern Province, Sri Lanka
Source: PLoS One. 2024 Jun 11;19(6):e0304690. doi: 10.1371/journal.pone.0304690 (PMC11166322; doi:10.1371/journal.pone.0304690)
Supplement: Attachment — Submitted filename: Reviewer Report for PLOS One 2024.docx [file pone.0304690.s001.docx]

**Reviewer's Comments to the Author**

Dear Editor,

In response to the review assignment for the manuscript PONE-D-23-38844 titled “**Prevalence and predictors of antibiotic prescription among patients hospitalized with Viral Lower Respiratory Tract Infections in Southern Province, Sri Lanka**”, kindly find my comments below:

Reviewer's Comments to the Author

The study investigated and attempted to identify the prevalence and predictors of antibiotic prescription among children and adults hospitalized with viral Lower Respiratory Tract Infections (LRTI) in a single tertiary health center in Sri Lanka.

The study focus and type were well within the scope of the journal.

The concept of the study is germane, and the expected contribution to knowledge is also good. The methods used were appropriate, while considerable analyses of collated data and relevant statistical analyses were done. The manuscript was also concisely written.

1. **Title**

The title is appropriate and manages to capture the essence of the study.

2. **Abstract**:
   The Abstract section was concise and well-written. The statistical methods employed were also included in the method segment of the abstract.
3. **The** **introduction**:

This section was generally well-written and captures the background of the study appropriately.

4. **Materials and Methods**:

This section was concisely written to allow for clear understanding and even reproducibility. The study design and sample size were adequate. Inclusion criteria for participants were clearly stated, and the structure of the questionnaire employed was highlighted.

5. **Results–**

The result section was explicit, and a lot of the data generated was highlighted, and comprehensively expressed in the result section. However, the statement “*this could be attributed to the early pandemic practices in Sri Lanka, where patients with COVID-19 were*

*being admitted to specialized isolation hospitals*.......” in lines 168 - 169 should be inserted in the appropriate section in the discussion segment.

Again, on line 178, the demographics of children and adult males were accounted for, but no mention was made of female participants. The demographic demarcation should be properly defined as children and adults, and all adult participants either male or female should be accounted for as adults.

6. **Discussion**

All inferences expressed under results were comprehensively addressed and discussed in the discussion section. The results were related appropriately to the discussion to capture the findings of the research work. The discussion was done vis-a-vis other related studies to reach a balanced conclusion. As it is, the results aligned completely with the discussion.

**Decision:** To accept after reviewing and addressing the issues raised. addressing the issues raised.
